# Phylogeography and resistome of pneumococcal meningitis in West Africa before and after vaccine introduction

**DOI:** 10.1099/mgen.0.000506

**Published:** 2021-07-30

**Authors:** Madikay Senghore, Peggy-Estelle Tientcheu, Archibald Kwame Worwui, Sheikh Jarju, Catherine Okoi, Sambou M. S. Suso, Ebenezer Foster-Nyarko, Chinelo Ebruke, Mohamadou Sonko, Mamdou Hama Kourna, Joseph Agossou, Enyonam Tsolenyanu, Lorna Awo Renner, Daniel Ansong, Bakary Sanneh, Catherine Boni Cisse, Angeline Boula, Berthe Miwanda, Stephanie W. Lo, Rebecca A. Gladstone, Stephanie Schwartz, Paulina Hawkins, Lesley McGee, Keith P. Klugman, Robert F. Breiman, Stephen D. Bentley, Jason M. Mwenda, Brenda Anna Kwambana-Adams, Martin Antonio

**Affiliations:** ^1^​ WHO Collaborating Centre for New Vaccines Surveillance, Medical Research Council Unit The Gambia at London School of Hygiene and Tropical Medicine, P.O. Box 273, Banjul, The Gambia; ^2^​ Center for Communicable Disease Dynamics, Harvard T.H. Chan School of Public Health, 677 Huntington Avenue, Boston, MA, USA; ^3^​ Hopital d’Enfants Albert Royer, BP 5297, Fann, Dakar, Senegal; ^4^​ Hospital National Niamey, BP 238, Niamey, Niger; ^5^​ Department of Mother and Child, Faculty of Medicine, University of Parakou, Parakou, Benin; ^6^​ Borgou Regional University Teaching Hospital, Parakou, Benin; ^7^​ Laboratoire Microbiologie, Centre Hospitalier Universitaire de Tokoin Lomé, BP 57, Lomé, Togo; ^8^​ Central Laboratory Services, Korle-Bu Teaching Hospital, P.O. Box 77, Accra, Ghana; ^9^​ Komfo Anokye Teaching Hospital, P.O. Box 1934, Kumasi, Ghana; ^10^​ Edward Francis Small Teaching Hospital, Banjul, The Gambia; ^11^​ Laboratoire Central du CHU de Yopougon, Institut Pasteur de Cote d'Ivoire, Abidjan, Ivory Coast; ^12^​ Centre Mere et Enfant de la Fondation, Chantal Biya, Yaounde, Cameroon; ^13^​ Institut National de Recherche Biomedicale, Kinshasa, Democratic Republic of Congo; ^14^​ Parasites and Microbes, Wellcome Sanger Institute, Hinxton, UK; ^15^​ Centers for Disease Control and Prevention, Atlanta, GA, USA; ^16^​ Rollins School of Public Health, Emory University, Atlanta, GA, USA; ^17^​ Emory Global Health Institute, Atlanta, GA, USA; ^18^​ World Health Organization Regional Office for Africa, BP 6, Brazzaville, Republic of Congo; ^19^​ NIHR Global Health Research Unit on Mucosal Pathogens, Division of Infection and Immunity, University College London, London, UK

**Keywords:** antibiotic resistance, genomic epidemiology, paediatric meningitis, pneumococcus, West and Central Africa

## Abstract

Despite contributing to the large disease burden in West Africa, little is known about the genomic epidemiology of *Streptococcus pneumoniae* which cause meningitis among children under 5 years old in the region. We analysed whole-genome sequencing data from 185 *S*. *pneumoniae* isolates recovered from suspected paediatric meningitis cases as part of the World Health Organization (WHO) invasive bacterial diseases surveillance from 2010 to 2016. The phylogeny was reconstructed, accessory genome similarity was computed and antimicrobial-resistance patterns were inferred from the genome data and compared to phenotypic resistance from disc diffusion. We studied the changes in the distribution of serotypes pre- and post-pneumococcal conjugate vaccine (PCV) introduction in the Central and Western sub-regions separately. The overall distribution of non-vaccine, PCV7 (4, 6B, 9V, 14, 18C, 19F and 23F) and additional PCV13 serotypes (1, 3, 5, 6A, 19A and 7F) did not change significantly before and after PCV introduction in the Central region (Fisher's test *P* value 0.27) despite an increase in the proportion of non-vaccine serotypes to 40 % (*n*=6) in the post-PCV introduction period compared to 21.9 % (*n*=14). In the Western sub-region, PCV13 serotypes were more dominant among isolates from The Gambia following the introduction of PCV7, 81 % (*n*=17), compared to the pre-PCV period in neighbouring Senegal, 51 % (*n*=27). The phylogeny illustrated the diversity of strains associated with paediatric meningitis in West Africa and highlighted the existence of phylogeographical clustering, with isolates from the same sub-region clustering and sharing similar accessory genome content. Antibiotic-resistance genotypes known to confer resistance to penicillin, chloramphenicol, co-trimoxazole and tetracycline were detected across all sub-regions. However, there was no discernible trend linking the presence of resistance genotypes with the vaccine introduction period or whether the strain was a vaccine or non-vaccine serotype. Resistance genotypes appeared to be conserved within selected sub-clades of the phylogenetic tree, suggesting clonal inheritance. Our data underscore the need for continued surveillance on the emergence of non-vaccine serotypes as well as chloramphenicol and penicillin resistance, as these antibiotics are likely still being used for empirical treatment in low-resource settings. This article contains data hosted by Microreact.

## Impact Statement

*Streptococcus pneumoniae*causes severe diseases including meningitis and pneumonia which are leading causes of morbidity and mortality in sub-Saharan Africa, particularly among children. Resource constraints make it difficult to carry out paediatric infectious disease surveillance in this setting. We compiled a genomic dataset of *S. pneumoniae* strains associated with paediatric bacterial meningitis in West and Central Africa, collected through sentinel surveillance. This unique dataset has allowed us to study the genetic determinants of antimicrobial resistance in the pre- and post-PCV introduction periods. We also show novel insights into the phylogenetic landscape of *S*. *pneumoniae* associated with paediatric meningitis and highlight patterns consistent with the localization of some sublineages by geographical sub-region.

## Data Summary

The sequencing reads for the genomes analysed have been deposited in the European Nucleotide Archive and the accession numbers for each isolate are listed in Table S1 (available with the online version of this article). A phylogenetic tree and associated metadata are available on Microreact: https://microreact.org/project/HJcM_9lxf.

**Impact Statement**

*Streptococcus pneumoniae* (the pneumococcus) is one of the top three causes of acute bacterial meningitis, the most severe form of meningitis [1]. Despite the availability of effective vaccines, acute bacterial meningitis due to the pneumococcus continues to be a major cause of morbidity and mortality in sub-Saharan Africa, especially among children under 5 years old [2]. The 10- or 13-valent pneumococcal conjugate vaccine (PCV10 and PCV13 respectively), which target the most prevalent serotypes in serious disease, have been introduced in 39/47 countries in sub-Saharan Africa. PCVs have been effective at reducing invasive disease caused by vaccine serotypes in high-income countries and low-resource settings [3–7].

Whole-genome sequencing has emerged as a useful technique for typing bacteria because it provides high-resolution inter-isolate comparisons, which can provide insights into pneumococcal transmission and evolution [8, 9]. For example, in 2016, 3 years post-PCV13 introduction, we identified a novel clade of sequence type (ST) 303 serotype 1 pneumococcus*,* which caused a meningitis outbreak in Ghana [10]. Access to genome sequencing and a well-defined global phylogeny of serotype 1 strains [11] allowed us to place the outbreak isolates in a global context. Unfortunately, in most West and Central African settings there remains a paucity of genomic data for pneumococcal serotypes associated with invasive disease.

An added advantage of genome sequencing is that it allows the prediction of resistance to commonly prescribed antibiotics [12]. For example, macrolide, chloramphenicol and tetracycline resistance can be predicted by the presence of the horizontally acquired *ermB* and *ormef, cat* and *tetM* genes, respectively [12–15]. Similarly, rifampicin, penicillin and co-trimoxazole resistance can be inferred based on known mutations in *rpoB,* penicillin binding protein genes *pbp1A*, *pbp2B* and *pbp2X*, and *folA/folP* genes, respectively [10, 12–16]. Through genomics, we can identify the emergence and spread of multidrug-resistant clones that could pose a serious public-health threat, especially in high disease burden settings.

We present genomic analysis of 185 pneumococcal isolates from children with confirmed meningitis in West Africa between 2010 and 2016. The collection of these isolates was facilitated through the World Health Organization (WHO) Invasive Bacterial Vaccine-Preventable Disease (IB-VPD) sentinel surveillance network. Our analysis probes the effect of PCV introduction on the genomic epidemiology of pneumococci causing paediatric meningitis. We also studied the potential effects of PCV introduction on the distribution of antibiotic-resistance genes. These data provide the first baseline data on the genomic epidemiology of pneumococcal paediatric meningitis in West Africa pre- and post-PCV introduction.

## Methods

### Study design

The WHO IB-VPD surveillance is supported by the WHO Collaborating Centre (WHO CC) for New Vaccines Surveillance hosted by the Medical Research Council Unit The Gambia at the London School of Hygiene and Tropical Medicine (MRCG). Within this framework, vaccine preventable meningitis surveillance has been on-going in 11 countries across 17 sentinel sites since 2010. As part of the surveillance, bacterial isolates recovered from suspected paediatric meningitis cases were sent to the WHO CC hosted at the MRCG for confirmation, serotyping, antibiotic-susceptibility testing and whole-genome sequencing in collaboration with the Global Pneumococcal Sequencing project (https://www.pneumogen.net/gps/).

### Study population

The study participants were aged between 6 days and 59 months old, the mean age was 20.8 months and the median age was 14 months (age was recorded for 129 patients). This dataset also included two older children aged 8 and 16 years old. Patients admitted with suspected meningitis at paediatric teaching and referral hospitals were enrolled into the surveillance. Isolates for whole-genome sequencing were received from sentinel sites in The Gambia, Senegal, Ivory Coast, Ghana, Togo, Benin, Niger, Cameroon and the Democratic Republic of Congo (Table 1).

**Table 1.** Counts of study isolates from each country by vaccine era

Sub-region	Country	PCV introduction*	Pre-PCV [*n* (%)]	Post-PCV [*n* (%)]	Unknown [*n* (%)]	Total
Eastern	Cameroon	2011	2 (6.9)	27 (93.1)	0 (0)	29
	DR Congo	2011	0 (0)	0 (0)	1 (100)	1
Central	Ghana	2012	17 (65.4)	9 (34.6)	0 (0)	26
	Benin	2011	3 (60)	2 (40)	0 (0)	5
	Ivory Coast	2014	2 (100)	0(0)	(0)	2
	Niger	2014	23 (92)	(0)	2 (8)	25
	Togo	2014	19 (82.6)	4 (17.4)	0 (0)	23
Western	Senegal	2013	53 (96.4)	2 (3.6)	0 (0)	55
	The Gambia	2009	0 (0)	19 (100)	0 (0)	19
Total	–	–	119 (64.3)	63 (34.1)	3 (1.6)	185

*Post-PCV period commences the year after the introduction of a PCV vaccine of any valency.

### Bacteriology

Lumbar puncture was performed to collect cerebrospinal fluid (CSF) specimens w. For some patients, specimens were also collected from other clinical sites, including blood, lung/pleural aspirate and pus. Our dataset includes isolates from CSF (151, 81.6 %), blood (21, 11.4 %), lung/pleural aspirate (9, 4.9 %), pus (1, 0.5 %) and 3 (1.6 %) from an unknown source. The pneumococcal isolates were cultured using standard techniques and sent to the WHO Regional Reference Laboratory (RRL) for further analysis.

Clinical specimens were streaked onto Columbia agar with 5 % defibrinated sheep blood and incubated overnight in 5 % CO_2_ at 37 °C. Suspected colonies of the pneumococcus were confirmed by sensitivity to optochin (Oxoid) and stored in 16 % (v/v) glycerol broth at −70 °C for shipment to the WHO RRL. Confirmation of species and characterization of serotypes were done by PCR and latex agglutination, as described elsewhere [16]. Antimicrobial susceptibility to cefotaxime, chloramphenicol, meropenem, vancomycin, co-trimoxazole, rifampicin, tetracycline, oxacillin (for penicillin) and erythromycin was performed by disc diffusion, and interpreted according to the Clinical and Laboratory Standards Institute (CLSI) guidelines. Confirmatory Etest was done for cefotaxime and ceftriaxone by colleagues at the US Centers for Disease Control and Prevention (CDC). All the disc diffusion assays were performed at the WHO RRL. Minimum inhibitory concentrations (MICs) could not be assessed due to the inhibitory cost of the kits. The WHO RRL participates in external quality assurance (EQA) programmes organized by the United Kingdom National External Quality Assessment Service (UK NEQAS), the National Institute for Communicable Diseases (NICD), South Africa, and the United States Centers for Disease Control and Prevention (US CDC).

### Whole-genome sequencing

Genomic DNA was extracted and purified from fresh overnight cultures of the stored pneumococcal isolates using a modified Qiagen extraction protocol as previously described [10]. Paired-end sequencing on the Illumina HiSeq platform was performed at the Wellcome Sanger Institute, Cambridge, UK [17].

### Genomic analysis

The *in silico* serotype and the multilocus sequence type (MLST) were determined from the genomes using SeroBA [18] and a local alignment of *de novo* contigs against MLST alleles in pubMLST [19], respectively. Sequencing reads were mapped to the pneumococcal strain ATCC 700669 reference genome (accession no. FM211187) using bwa (version 0.7.17) with default parameters [20], the bam files were sorted and duplicates were marked using Picard. The mpileup command in SAMtools (version 1.2.1) was used to call bases at all sites with at least five reads mapped, and output the calls in the variant call format (VCF) [21]. A consensus sequence was generated and SNPs were called where at least 75 % of reads mapped to the alternative allele. The consensus sequences from all genomes were amalgamated into a multiple sequence alignment fasta file and variable sites were extracted using the SNP-sites program (version 2.5.1) [22]. The maximum-likelihood phylogeny was reconstructed from 99 474 variable sites in the core genome using RAxML (version 8.2.8) with a general time reversible model and 100 bootstrap replicates [23]. The phylogenetic tree and associated metadata are publicly available on Microreact: https://microreact.org/project/HJcM_9lxf.

*De novo* assemblies were generated from sequencing using The Sanger Institute assembly pipeline that is described elsewhere [24]. Assemblies were annotated using Prokka (version 1.14.5) [25] and the pan genome was inferred using Roary (version 3.12) [26]. Genomes were clustered based on the presence and absence of accessory genes using panini [27] and visualized in a scatter plot using Microreact. panini uses a Student's *t*-distributed stochastic neighbour embedding machine learning algorithm to compute similarities in the accessory genome. Antimicrobial resistance was predicted based on the presence of known genotypes at loci that are associated with antibiotic resistance (*rpoB*, *pbp1A*, *pbp2B*, *pbp2B* and *folA/folP* genes) using an antibiotic-resistance calling pipeline designed for *S. pneumoniae* [12, 28–31]. The *ermB*/*mefA*, *cat* and *tetM* genes were linked to erythromycin, chloramphenicol and tetracycline resistance, respectively. Genotypic variants of *pbp* and *folA/folP* genes known to confer resistance were attributed to penicillin and co-trimoxazole resistance, respectively.

### Statistical analysis

Data were compiled in Excel and uploaded onto RStudio (version 1.2.5033) for statistical analysis. The analyses were carried out separately on genomes from the Western and Central sub-regions. Paired *t*-test, chi-square (χ^2^) test and Fisher’s exact test were used to analyse the differences in the distribution of vaccine and non-vaccine serotypes in the pre- and post-PCV introduction periods. There were only two isolates from the pre-PCV period in the Eastern sub-region, so no comparisons could be made.

## Results

We analysed 185 genomes of pneumococci isolated from paediatric meningitis patients across West Africa and parts of Central Africa. The isolates were clustered based on country into three sub-regions: isolates from Gambia and Senegal were clustered as the Western sub-region; Ghana, Togo, Ivory Coast, Benin and Niger were clustered as the Central West sub-region; and Cameroon and the Democratic Republic of Congo represented the Eastern sub-region. We defined the pre-PCV period as the time period preceding the first introduction of the PCV (any valency) in a given country and post-PCV as the time post-introduction of PCV. A third of the isolates were recovered from cases that occurred post-PCV introduction and two thirds were pre-PCV (Table 1).

### Serotype distribution across sub-regions

**Fig. 1.**
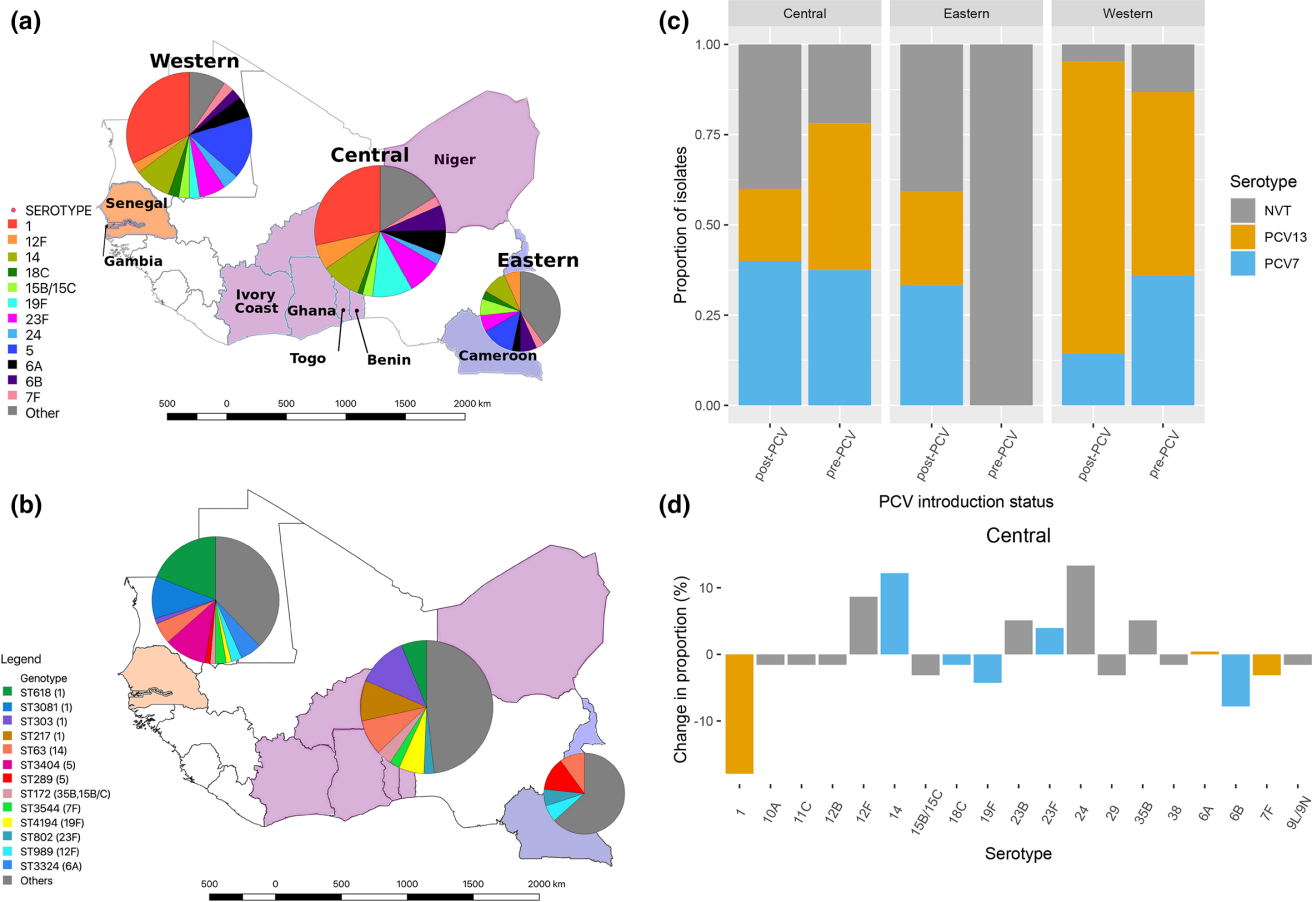
Distribution of the major *S. pneumoniae* serotypes and genotypes among isolates from suspected paediatric meningitis cases pre- and post-PCV introduction in West and Central Africa grouped by sub-region. (**a**) A map of West Africa including Cameroon with pie charts showing the distribution of the main serotypes. (**b**) A map with pie charts showing the distribution of the main STs. (**c**) A stacked column plot showing the proportion of isolates bearing non-vaccine serotypes, PCV7 serotypes and additional PCV13 serotypes before and after the introduction of PCV. (**d**) A bar graph showing the change in prevalence of the most common serotypes before and after PCV introduction in the Western and Central sub-regions.

We classified our strains into serotypes and MLSTs based on the whole genome, and visualized their distribution in the three sub-regions as a means of studying sub-regional diversity. There were 32 serotypes identified among the cases, which included 76 unique Sequence Types (STs). Most isolates belonged to serotypes 1 (47, 25.4 %), 14 (18, 9.7 %), 5 (16, 8.6 %) and 23F (14, 7.6 %). Other commonly identified serotypes included 19F (10, 5.4 %), 6A (10, 5.4 %), 12F (9, 4.9 %), 6B (9, 4.9 %) and 15B/15C (6, 3.2 %). Serotype 1 was dominant in the West and Central sub-regions, but it was not detected in the Eastern sub-region (Fig. 1a, b). Most serotypes were uncommon (20, 62.5 %) and were only detected in one or two patients (Fig. 1a).

In the Central sub-region, isolates were sequenced from 64 and 15 patients in the pre-and post-PCV introduction periods, respectively. The overall distribution of non-vaccine serotypes, PCV7 serotypes (4, 6B, 9V, 14, 18C, 19F and 23F) and additional PCV13 serotypes (1, 3, 5, 6A, 19A and 7F) did not change significantly before and after PCV introduction in the Central region: the proportion of non-vaccine serotypes in the post-PCV period was 40 % (*n*=6) compared to the pre-PCV period, 21.9 % (*n*=14) (Fisher test *P* value 0.27) (Fig. 1c). The proportion of isolates with the non-vaccine serotypes 24, 12F, 35B and 23B increased by 13.3, 8.7, 6.3 and 5.1 %, respectively, in the post-PCV period (Fig. 1d, Table S2). In the Western sub-region, all pre-PCV isolates were from Senegal (*n*=53), while 90 % (*n*=19) of the post-PCV introduction isolates were from The Gambia after the introduction of PCV7, which precluded a comparison based on the PCV era.

### Phylogenetic analysis and sub-regional clustering

**Fig. 2.**
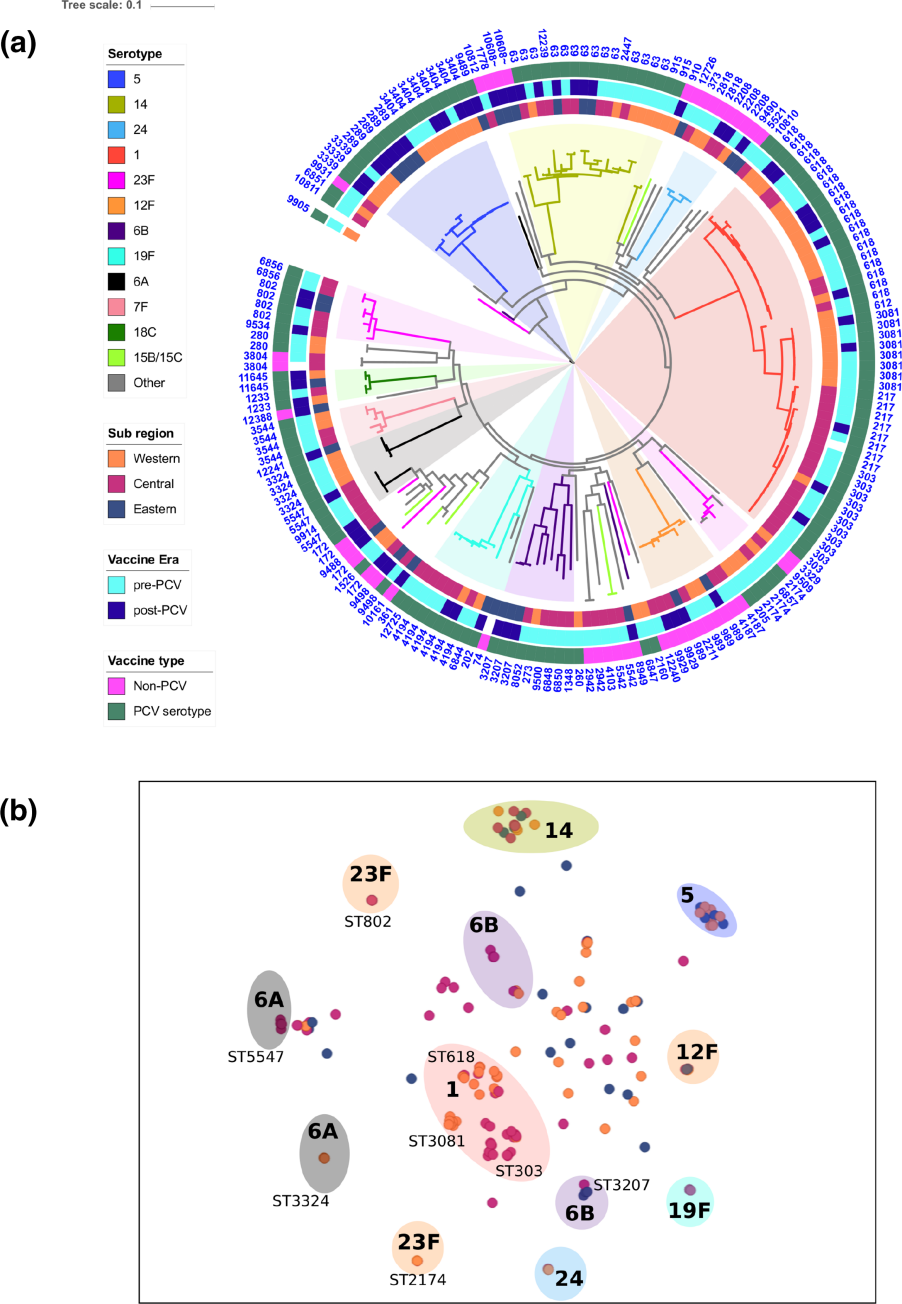
Phylogeny of *S. pneumoniae* genotypes causing paediatric meningitis in West and Central Africa, and a scatter plot showing accessory genome similarity. (**a**) A phylogenetic tree annotated with branches coloured by serotype, with metadata rings to show sub-region of origin, vaccine era and vaccine type with ST displayed as text on the outer ring. (**b**) A panini accessory genome scatter plot where each point, representing one isolate, is coloured by serotype, and distances between points are proportional to accessory genome similarity. The panini plot is by major serotypes (in bold) and the STs that demonstrate geographical clustering.

The phylogenetic tree and a scatter plot depicting similarity of accessory genomes content were annotated with sub-region and serotype in order to visualize potential clustering of strains from the same sub-region. Phylogeographical clustering was observed within serotypes, with isolates from the same sub-region clustering and sharing similar accessory genome content (Fig. 2a). In West Africa, sub-regional clustering was observed within the serotype 1 clade: ST3081 was found only in the Western sub-region, while ST303 was the predominant genotype in the Central sub-region. Serotype 6A isolates formed two divergent clades with markedly different accessory genome content. The ST3324 subclade was prevalent in the Western sub-region and a ST5547 subclade was prevalent in the Central sub-region.

Serotype 5 was not found in the Central sub-region, but in the Western sub-region it was the second most common serotype. Three unique STs were present in the Western sub-region, but in the Eastern sub-region only ST289 was reported. Serotypes 5, 12F, 14 and 19F had a highly conserved serotype specific accessory genome and did not vary with ST or sub-region of origin (Fig. 2b).

**Fig. 3.**
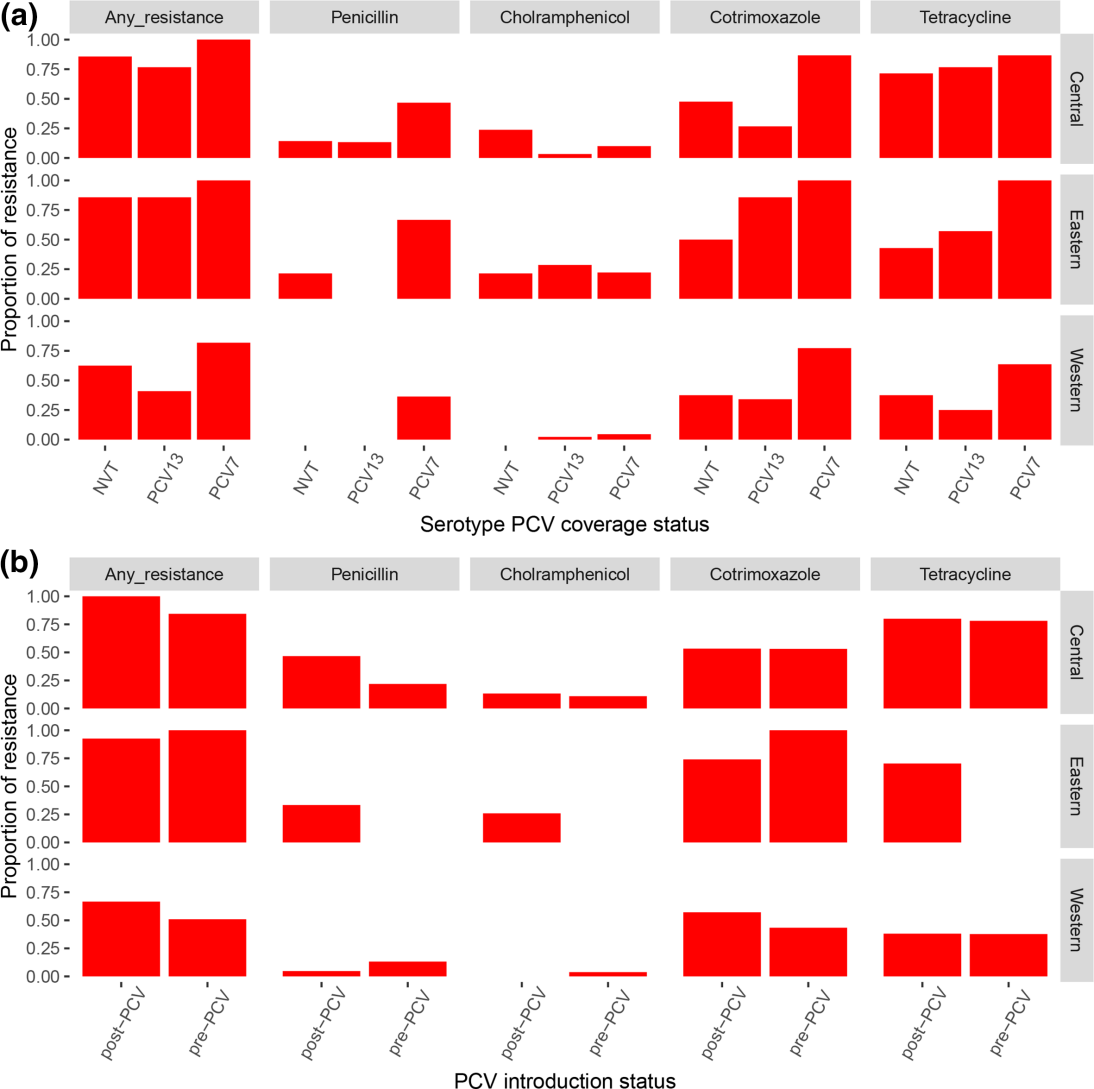
Trends in the presence and absence of antibiotic-resistance genotypes in the context of sub-region and PCV introduction period. (**a**) A column plot showing the proportion of genomes bearing antibiotic-resistance genes among serotypes, which were grouped according to whether they were PCV7 serotypes, additional PCV13 serotypes or non-vaccine serotypes. (**b**) A column plot showing the proportion of isolates bearing antibiotic-resistance among isolates from the pre-and post-PCV introduction periods in each sub-region. Note that only two isolates from the Eastern sub-region in the pre-PCV introduction period were available.

**Fig. 4.**
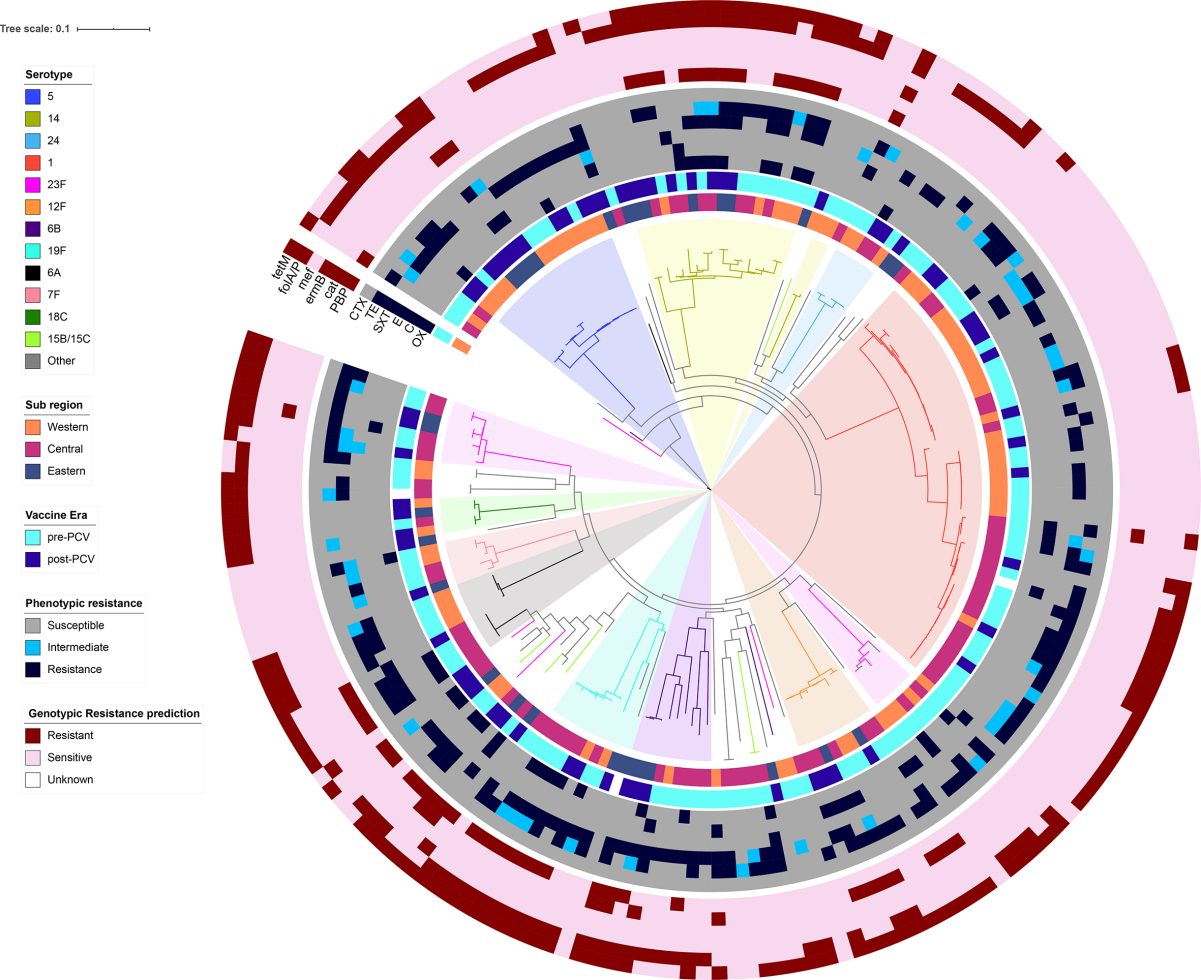
Antibiotic resistance and resistance genotype patterns in the context of the whole genome phylogeny. A phylogenetic tree with branches coloured by serotype and metadata blocks corresponding to sub region, vaccine introduction period, phenotypic antibiotic resistance patterns and presence of antibiotic resistance genes for penicillin (*PBP*, OX), chloramphenicol (*cat*, C), erythromycin (*mef/ermB*, E), co-trimoxazole (*folP/ folA*, SXT), tetracycline (*tetM*, TE) and cefotaxime (CTX).

### Resistance genotypes in the context of sub-region, PCV introduction period and phylogeny

Our data confirm the presence of antibiotic-resistance genotypes known to confer resistance to penicillin (*n*=38, 20.5 %), chloramphenicol (*n*=18, 9.7 %), co-trimoxazole (*n*=101, 54.6 %), erythromycin (*n*=3, 1.6 %) and tetracycline (*n*=111, 60 %). There did not appear to be a significant correlation between the presence of antibiotic resistance and sub-region (Fig. 3). Likewise, there was no significant trend linking the presence of resistance genotypes to the vaccine introduction period or whether the strain was a vaccine or non-vaccine serotype (Fig. 3). The genotypic predictors of resistance among the isolates were examined in the context of the phylogeny. Resistance genotypes appeared to be conserved within selected sub-clades of the phylogenetic tree (Fig. 4). Within serotype 1, isolates harbouring both *tetM* and *folA/ folP* resistance genotypes were common in the ST303/ST217 sub-clade (*n*=8, 42 %) but absent in the ST3081 (*n*=0), and only four isolates in the ST618 sub-clade (20 %) bore the *tetM* resistance gene (Fig. 4). Prediction of antimicrobial susceptibility from the genome has been found to have good correlation with phenotypic data [12]. However, we noted 23 (12.4 %) chloramphenicol-resistant cases but the *cat* gene was only detected in 13 (56.3 %) cases. Similarly, among 33 penicillin-resistant cases, 24 (72.7 %) had a *pbp* genotype known to confer resistance. Unfortunately, we were unable to perform phenotypic antimicrobial susceptbility retesting to confirm the results.

## Discussion

Our dataset offers important insights into the genomic epidemiology of *S. pneumoniae* associated with meningitis in West and Central Africa, a high-burden region with a paucity of genomic and epidemiological data. Phylogeographical clusters of isolates causing meningitis in the same geographical sub-region shared similar accessory genome content. The pneumococcus has an open pan genome that can readily acquire accessory genes from microbes within its ecological niche, making it highly adaptable to its environment [32, 33]. The accessory genome content plays a major role in determining the fitness of pneumococcal lineages and re-shaping the perturbed bacterial population structure following major clinical interventions like vaccination [34, 35]. Further work needs to be done to determine whether children in some parts of West and Central Africa are at a greater risk of developing meningitis due to the presence of virulent pneumococcal lineages.

Although our dataset was not powered to study the impact of PCV on the epidemiology of pneumococcal bacterial meningitis among children, we gleaned some observations on the distribution of serotypes before and after the introduction of PCV. Serotype 1 remained a leading cause of paediatric pneumococcal meningitis. In the Central sub-region, the prevalence of serotype 1 decreased by 17.9 % following the introduction of PCV13. Serotype 1 has been causing invasive disease for a long time and has evolved into distinct lineages that have adapted to specific geographical localities [11, 36]. In sub-Saharan Africa, serotype 1 encompasses highly virulent clones, some of which are capable of causing lethal outbreaks of meningitis [10, 37–39].

The high burden of serotype 1 in this sub-region may contribute to the lag in decreasing the burden of serotype 1 disease in the post-PCV13 period. In Ghana, where a three-dose regimen of PCV13 was introduced without a booster, serotype 1 remained a common cause of invasive pneumococcal disease up to 3 years after the introduction of PCV13, and in that period caused a meningitis outbreak among older children and adults in Ghana [7, 10, 40]. Experts have previously suggested that a booster may be effective in expediting the decline of vaccine serotypes in sub-Saharan Africa [41]. In South Africa, a three-dose regimen with a booster dose was effective in rapidly decreasing the burden of PCV13 serotypes, including serotype 1 [42]. A nuanced analysis of the role of boosters is needed based on robust epidemiological data or a clinical trial.

The replacement of vaccine serotypes causing invasive diseases by non-vaccine serotypes has been reported to various extents in several countries following the introduction of PCVs [7, 43–46]. The Global Pneumococcal Sequencing consortium has identified serotype 12F among the top five leading serotypes of the post-PCV13 period in at least two different countries [47]. In the Central sub-region, the prevalence of non-PCV serotypes, including serotype 12F, increased following the introduction of PCV13 serotypes. This underscores the need for continued surveillance in West Africa, to carefully monitor the role of non-vaccine serotypes in the post-PCV13 introduction period.

Antibiotic resistance can contribute to treatment failures and negatively impact clinical outcomes [48]. There was no clinically defined resistance to cefotaxime and ceftriaxone, which are the primary WHO-recommended antibiotics [49]. Isolates bearing the penicillin-resistance *pbp* genotypes were recovered from all sub-regions; this is an important finding because 49 when neither cefotaxime nor ceftriaxone are available, penicillin may be used to treat meningitis.

Championing improved infection management, diagnosis and antibiotic stewardship in sub-Saharan Africa may curb the emergence and spread of paediatric pneumococcal infections that are resistant to the recommended drugs [50–52]. This is challenging to implement in a low-resource setting with infrastructural constraints; and warrants special attention [53]. Vaccines have been proposed as a potential mechanism for reducing the burden of antibiotic resistance by lowering the risk of infection [54]. This was not reflected in this dataset, as a decrease in the prevalence of antibiotic-resistance genes was not associated with the post-PCV introduction era, and there were no discernible trends regarding the presence of resistance genotypes in vaccine and non-vaccine serotypes. While this may be reassuring, continued genomic surveillance is required to monitor these trends and to understand the underlying evolutionary mechanisms. We may be missing the contribution of novel resistance mechanisms that have not been discovered yet and resistance mechanisms that are difficult to predict with whole-genome sequencing, such as efflux pump upregulation and cell wall permeability changes [55, 56]. Additionally, our dataset may be affected by the use of antibiotic prior to hospital admission.

### Conclusion

Our study underscores the need for continued surveillance for monitoring the emergence of non-vaccine serotypes as well as residual serotype 1. Genomic surveillance of pneumococcal disease and carriage in the African meningitis belt may provide an evidence-base to inform future strategies to enhance the control of the pneumococcus. Likewise, monitoring of antimicrobial resistance using genomic approaches may enhance the detection of drug resistant strains and contribute to ensuring that patients receive optimal treatment in low-resource settings.

### Study limitations

A limitation of this study is that PCVs were introduced in the countries which participated in the surveillance at different time points and the countries did not use the same PCV formulations i.e. PCV10 or PCV13. This made it difficult to get a representative subset of pre- and post-PCV isolates across all three sub-regions. The number of isolates recovered from some countries was very low, e.g. there were single isolates from Ivory Coast and Democratic Republic of Congo. Grouping the isolates into sub-regions made the analysis possible, but this introduced potential bias as serotype distributions likely differ between and within countries. Furthermore, a limitation of sentinel surveillance is that we likely missed a significant proportion of pneumococcal meningitis cases that did not attend the sentinel sites. This is further compounded by the low culture-recovery rates for the pneumococcus across the sub-region. Coordinating surveillance in a low-resource setting is extremely challenging. This dataset highlights a need to develop laboratory capacity in this sub-Saharan Africa.The inability to perform retrospective confirmatory MIC testing may have potentially contributed to the discrepancies between MICs and genomic predictions.

## Supplementary Data

Supplementary material 1

## References

[1] Kim KS (2010). Acute bacterial meningitis in infants and children. Lancet Infect Dis.

[2] Lukšić I, Mulić R, Falconer R, Orban M, Sidhu S (2013). Estimating global and regional morbidity from acute bacterial meningitis in children: assessment of the evidence. Croat Med J.

[3] Cohen R, Levy C (2017). 13-valent pneumococcal conjugate vaccine in Africa. Lancet Glob Health.

[4] Moore MR, Link-Gelles R, Schaffner W, Lynfield R, Holtzman C (2016). Effectiveness of 13-valent pneumococcal conjugate vaccine for prevention of invasive pneumococcal disease in children in the USA: a matched case-control study. Lancet Respir Med.

[5] Miller E, Andrews NJ, Waight PA, Slack MPE, George RC (2011). Effectiveness of the new serotypes in the 13-valent pneumococcal conjugate vaccine. Vaccine.

[6] Cohen C, von Mollendorf C, de Gouveia L, Lengana S, Meiring S (2017). Effectiveness of the 13-valent pneumococcal conjugate vaccine against invasive pneumococcal disease in South African children: a case-control study. Lancet Glob Health.

[7] Mackenzie GA, Hill PC, Jeffries DJ, Hossain I, Uchendu U (2016). Effect of the introduction of pneumococcal conjugate vaccination on invasive pneumococcal disease in The Gambia: a population-based surveillance study. Lancet Infect Dis.

[8] Croucher NJ, Harris SR, Grad YH, Hanage WP (2013). Bacterial genomes in epidemiology – present and future. Philos Trans R Soc Lond B Biol Sci.

[9] Parkhill J, Wren BW (2011). Bacterial epidemiology and biology-lessons from genome sequencing. Genome Biol.

[10] Kwambana-Adams BA, Asiedu-Bekoe F, Sarkodie B, Afreh OK, Kuma GK (2016). An outbreak of pneumococcal meningitis among older children (≥5 years) and adults after the implementation of an infant vaccination programme with the 13-valent pneumococcal conjugate vaccine in Ghana. BMC Infect Dis.

[11] Cornick JE, Chaguza C, Harris SR, Yalcin F, Senghore M (2015). Region-specific diversification of the highly virulent serotype 1 *Streptococcus pneumoniae*. Microb Genom.

[12] Metcalf BJ, Chochua S, Gertz RE, Li Z, Walker H (2016). Using whole genome sequencing to identify resistance determinants and predict antimicrobial resistance phenotypes for year 2015 invasive pneumococcal disease isolates recovered in the United States. Clin Microbiol Infect.

[13] Okitsu N, Kaieda S, Yano H, Nakano R, Hosaka Y (2005). Characterization of ermB gene transposition by Tn1545 and Tn917 in macrolide-resistant *Streptococcus pneumoniae* isolates. J Clin Microbiol.

[14] Cornick JE, Bentley SD (2012). *Streptococcus pneumoniae*: the evolution of antimicrobial resistance to beta-lactams, fluoroquinolones and macrolides. Microbes Infect.

[15] Ayoubi P, Kilic AO, Vijayakumar MN, Tn VMN (1991). Tn5253, the pneumococcal omega (cat tet) BM6001 element, is a composite structure of two conjugative transposons, Tn5251 and Tn5252. J Bacteriol.

[16] Hill PC, Cheung YB, Akisanya A, Sankareh K, Lahai G (2008). Nasopharyngeal carriage of *Streptococcus pneumoniae* in Gambian infants: a longitudinal study. Clin Infect Dis.

[17] Gladstone RA, Lo SW, Lees JA, Croucher NJ, van Tonder AJ (2019). International genomic definition of pneumococcal lineages, to contextualise disease, antibiotic resistance and vaccine impact. EBioMedicine.

[18] Epping L, van Tonder AJ, Gladstone RA, Bentley SD, The Global Pneumococcal Sequencing Consortium (2018). SeroBA: rapid high-throughput serotyping of *Streptococcus pneumoniae* from whole genome sequence data. Microbial Genomics.

[19] Page AJ, Taylor B, Keane J (2016). Multilocus sequence typing by blast from de novo assemblies against PubMLST. J Open Res Softw.

[20] Li H, Durbin R (2009). Fast and accurate short read alignment with Burrows-Wheeler transform. Bioinformatics.

[21] Li H, Handsaker B, Wysoker A, Fennell T, Ruan J (2009). The Sequence Alignment/Map format and SAMtools. Bioinformatics.

[22] Page AJ, Taylor B, Delaney AJ, Soares J, Seemann T (2016). *SNP-sites*: rapid efficient extraction of SNPs from multi-FASTA alignments. Microb Genom.

[23] Stamatakis A (2014). RAxML version 8: a tool for phylogenetic analysis and post-analysis of large phylogenies. Bioinformatics.

[24] Page AJ, De Silva N, Hunt M, Quail MA, Parkhill J (2016). Robust high-throughput prokaryote *de novo* assembly and improvement pipeline for Illumina data. Microb Genom.

[25] Seemann T (2014). Prokka: rapid prokaryotic genome annotation. Bioinformatics.

[26] Page AJ, Cummins CA, Hunt M, Wong VK, Reuter S (2015). Roary: rapid large-scale prokaryote pan genome analysis. Bioinformatics.

[27] Abudahab K, Prada JM, Yang Z, Bentley SD, Croucher NJ (2019). PANINI: pangenome neighbour identification for bacterial populations. Microb Genom.

[28] Metcalf BJ, Gertz RE, Gladstone RA, Walker H, Sherwood LK (2016). Strain features and distributions in pneumococci from children with invasive disease before and after 13-valent conjugate vaccine implementation in the USA. Clin Microbiol Infect.

[29] Li Y, Metcalf BJ, Chochua S, Li Z, Gertz RE (2016). Penicillin-binding protein transpeptidase signatures for tracking and predicting β-lactam resistance levels in *Streptococcus pneumoniae*. mBio.

[30] Li Y, Metcalf BJ, Chochua S, Li Z, Gertz RE (2017). Validation of β-lactam minimum inhibitory concentration predictions for pneumococcal isolates with newly encountered penicillin binding protein (PBP) sequences. BMC Genomics.

[31] Hunt M, Mather AE, Sánchez-Busó L, Page AJ, Parkhill J (2017). ARIBA: rapid antimicrobial resistance genotyping directly from sequencing reads. Microb Genom.

[32] Muzzi A, Donati C (2011). Population genetics and evolution of the pan-genome of *Streptococcus pneumoniae*. Int J Med Microbiol.

[33] Donati C, Hiller NL, Tettelin H, Muzzi A, Croucher NJ (2010). Structure and dynamics of the pan-genome of *Streptococcus pneumoniae* and closely related species. Genome Biol.

[34] Corander J, Fraser C, Gutmann MU, Arnold B, Hanage WP (2017). Frequency-dependent selection in vaccine-associated pneumococcal population dynamics. Nat Ecol Evol.

[35] Azarian T, Grant LR, Arnold BJ, Hammitt LL, Reid R (2018). The impact of serotype-specific vaccination on phylodynamic parameters of *Streptococcus pneumoniae* and the pneumococcal pan-genome. PLoS Pathog.

[36] Chaguza C, Cornick JE, Harris SR, Andam CP, Bricio-Moreno L (2016). Understanding pneumococcal serotype 1 biology through population genomic analysis. BMC Infect Dis.

[37] Williams TM, Loman NJ, Ebruke C, Musher DM, Adegbola RA (2012). Genome analysis of a highly virulent serotype 1 strain of *Streptococcus pneumoniae* from West Africa. PLoS One.

[38] Bricio-Moreno L, Ebruke C, Chaguza C, Cornick J, Kwambana-Adams B (2017). Comparative genomic analysis and in vivo modeling of *Streptococcus pneumoniae* ST3081 and ST618 isolates reveal key genetic and phenotypic differences contributing to clonal replacement of serotype 1 in The Gambia. J Infect Dis.

[39] Leimkugel J, Adams Forgor A, Gagneux S, Pflüger V, Flierl C (2005). An outbreak of serotype 1 *Streptococcus pneumoniae* meningitis in northern Ghana with features that are characteristic of *Neisseria meningitidis* meningitis epidemics. J Infect Dis.

[40] Bozio CH, Abdul-Karim A, Abenyeri J, Abubakari B, Ofosu W (2018). Continued occurrence of serotype 1 pneumococcal meningitis in two regions located in the meningitis belt in Ghana five years after introduction of 13-valent pneumococcal conjugate vaccine. PLoS One.

[41] Klugman KP, Madhi SA, Adegbola RA, Cutts F, Greenwood B (2011). Timing of serotype 1 pneumococcal disease suggests the need for evaluation of a booster dose. Vaccine.

[42] von Gottberg A, de Gouveia L, Tempia S, Quan V, Meiring S (2014). Effects of vaccination on invasive pneumococcal disease in South Africa. N Engl J Med.

[43] Ouldali N, Levy C, Varon E, Bonacorsi S, Béchet S (2018). Incidence of paediatric pneumococcal meningitis and emergence of new serotypes: a time-series analysis of a 16-year French national survey. Lancet Infect Dis.

[44] Ladhani SN, Collins S, Djennad A, Sheppard CL, Borrow R (2018). Rapid increase in non-vaccine serotypes causing invasive pneumococcal disease in England and Wales, 2000-17: a prospective national observational cohort study. Lancet Infect Dis.

[45] Ben-Shimol S, Givon-Lavi N, Grisaru-Soen G, Megged O, Greenberg D (2018). Comparative incidence dynamics and serotypes of meningitis, bacteremic pneumonia and other-IPD in young children in the PCV era: insights from Israeli surveillance studies. Vaccine.

[46] Weinberger R, von Kries R, van der Linden M, Rieck T, Siedler A (2018). Invasive pneumococcal disease in children under 16 years of age: Incomplete rebound in incidence after the maximum effect of PCV13 in 2012/13 in Germany. Vaccine.

[47] Lo SW, Gladstone RA, van Tonder AJ, Lees JA, du Plessis M (2019). Pneumococcal lineages associated with serotype replacement and antibiotic resistance in childhood invasive pneumococcal disease in the post-PCV13 era: an international whole-genome sequencing study. Lancet Infect Dis.

[48] Klugman KP (2007). Clinical impact of antibiotic resistance in respiratory tract infections. Int J Antimicrob Agents.

[49] World Health Organization (2013). Pocket Book of Hospital Care for Children.

[50] Kariuki S, Dougan G (2014). Antibacterial resistance in sub-Saharan Africa: an underestimated emergency. Ann N Y Acad Sci.

[51] Kimang'a AN (2012). A situational analysis of antimicrobial drug resistance in Africa: are we losing the battle?. Ethiop J Health Sci.

[52] Williams PCM, Isaacs D, Berkley JA (2018). Antimicrobial resistance among children in sub-Saharan Africa. Lancet Infect Dis.

[53] Essack SY, Desta AT, Abotsi RE, Agoba EE (2017). Antimicrobial resistance in the WHO African region: current status and roadmap for action. J Public Health.

[54] Klugman KP, Black S (2018). Impact of existing vaccines in reducing antibiotic resistance: primary and secondary effects. Proc Natl Acad Sci USA.

[55] Frimodt-Møller J, Løbner-Olesen A (2019). Efflux-pump upregulation: from tolerance to high-level antibiotic resistance?. Trends Microbiol.

[56] Pulzova L, Navratilova L, Comor L (2017). Alterations in outer membrane permeability favor drug-resistant phenotype of *Klebsiella pneumoniae*. Microb Drug Resist.

